# Online education resources for antimicrobial stewardship in Ethiopia: an educational resource review

**DOI:** 10.1093/jacamr/dlag193

**Published:** 2026-08-28

**Authors:** Mikiyas G Teferi, Kebron W Aweke, Gelila S Kassa, Selamawit Z Tadesse, Elias Gashaw Endegnanew, Eden Haile Hagos, Biruk T Mengistie, Chernet T Mengistie, Demmelash Gezahegn Nigatu, Eskedar Ferdu Azerefegne

**Affiliations:** School of Medicine, College of Health Sciences, Addis Ababa University, Addis Ababa, Ethiopia; Department of Family Medicine, Atrium Health Navicent, Mercer University, Macon, GA, USA; School of Medicine, College of Health Sciences, Addis Ababa University, Addis Ababa, Ethiopia; School of Medicine, College of Health Sciences, Addis Ababa University, Addis Ababa, Ethiopia; School of Medicine, College of Health Sciences, Addis Ababa University, Addis Ababa, Ethiopia; School of Medicine, College of Health Sciences, Addis Ababa University, Addis Ababa, Ethiopia; School of Medicine, College of Health Sciences, Addis Ababa University, Addis Ababa, Ethiopia; School of Medicine, College of Health Sciences, Addis Ababa University, Addis Ababa, Ethiopia; School of Medicine, College of Health Sciences, Addis Ababa University, Addis Ababa, Ethiopia; Department of Emergency Medicine and Critical Care, School of Medicine, College of Health Sciences, Addis Ababa University, Addis Ababa, Ethiopia; Infectious Disease Unit, Department of Internal Medicine, College of Health Sciences, Addis Ababa University, Addis Ababa, Ethiopia

## Abstract

**Background:**

Antimicrobial stewardship (AMS) in Ethiopia requires practical and scalable educational resources that can support healthcare workers in resource-limited settings. Online resources may help strengthen stewardship knowledge and implementation, but their value depends on accessibility, contextual relevance and practical utility.

**Objectives:**

To identify and appraise online AMS educational resources relevant to Ethiopia and to assess their potential contribution to AMS education and implementation.

**Methods:**

We conducted a targeted education resource review of six publicly accessible online AMS resources. Targeted searches combined antimicrobial stewardship (AMS) and antimicrobial resistance (AMR) education terms with Ethiopia, low- and middle-income country settings, toolkit, training, online course, massive open online course (MOOC), OpenWHO, FutureLearn, CDC, BSAC and WHO. Resources were evaluated for accessibility, educational design, contextual appropriateness, implementation utility and complementarity.

**Results:**

The Ethiopian national practical guide had the strongest implementation relevance because of its close alignment with local stewardship priorities and facility-level needs. WHO- and course-based resources added structured learning and broader programmatic support. Across resources, common limitations included dependence on internet connectivity, limited offline usability and insufficient locally contextualized case-based learning.

**Conclusions:**

The included resources offer complementary value for AMS education in Ethiopia, but no single resource is sufficient on its own. A blended, context-adapted approach that integrates national guidance with selected online educational resources is likely to provide the greatest educational and system-level benefit.

## Introduction

Antimicrobial stewardship (AMS) is central to global antimicrobial resistance (AMR) response strategies. The WHO global action plan defines five strategic objectives, including optimizing antimicrobial use, and provides a framework for national action plans.^1^ The Ethiopian Ministry of Health practical guide places stewardship within this broader architecture, stating that Ethiopia launched a third strategic plan for AMR prevention and containment for 2021 to 2025, aligned with the Health Sector Transformation Plan II, and describing AMS as an instrument for implementing the fourth strategic objective focused on optimizing antimicrobial use through stewardship practices.^1,2^

The educational problem is not simply awareness. The Ethiopian Ministry of Health practical guide cites a national point prevalence survey showing no functional AMS programmes in health care facilities and high inpatient antibiotic use (63.8%), with healthcare-associated infections as the most common indication category.^2,3^ This finding implies that stewardship education must be implementation-focused and competency-based: building facility governance, practical prescribing review workflows, audit and feedback cycles and pragmatic antibiotic selection tools such as the WHO Access, Watch and Reserve (AWaRe) antibiotic classification, which groups antibiotics to guide stewardship and access decisions, and IV-to-oral switching.^4,5^

Online educational resources can fill workforce development gaps, but their value depends on deliverability and context. In low- and middle-income country (LMIC) settings, common barriers to antimicrobial stewardship programme (ASP) development include limited diagnostics, limited drug availability, resistance to practice change and limited familiarity with stewardship concepts, which supports the need for practical, locally adaptable education rather than purely theoretical modules.^6^ Evidence also suggests that education alone can improve AMS in some contexts, but that sustained impact is harder to guarantee without mechanisms that embed change into practice, which reinforces the importance of pairing education with tools, measurement and governance structures.^7^

This education resource review therefore evaluates six online resources on AMS relevant to Ethiopia, using an appraisal framework that emphasizes both learning quality and implementation utility, and explicitly considers how each resource could integrate with Ethiopia’s national AMS strategy and workforce development.

## Methods

### Review format and reporting approach

This education resource review was prepared in line with JAC-AMR guidance for reviews of online educational resources. BSAC educational resource classification scheme was used to organize extracted metadata, including duration, access, language, accreditation, downloadability, resource type and applicability.

**Figure 1.**
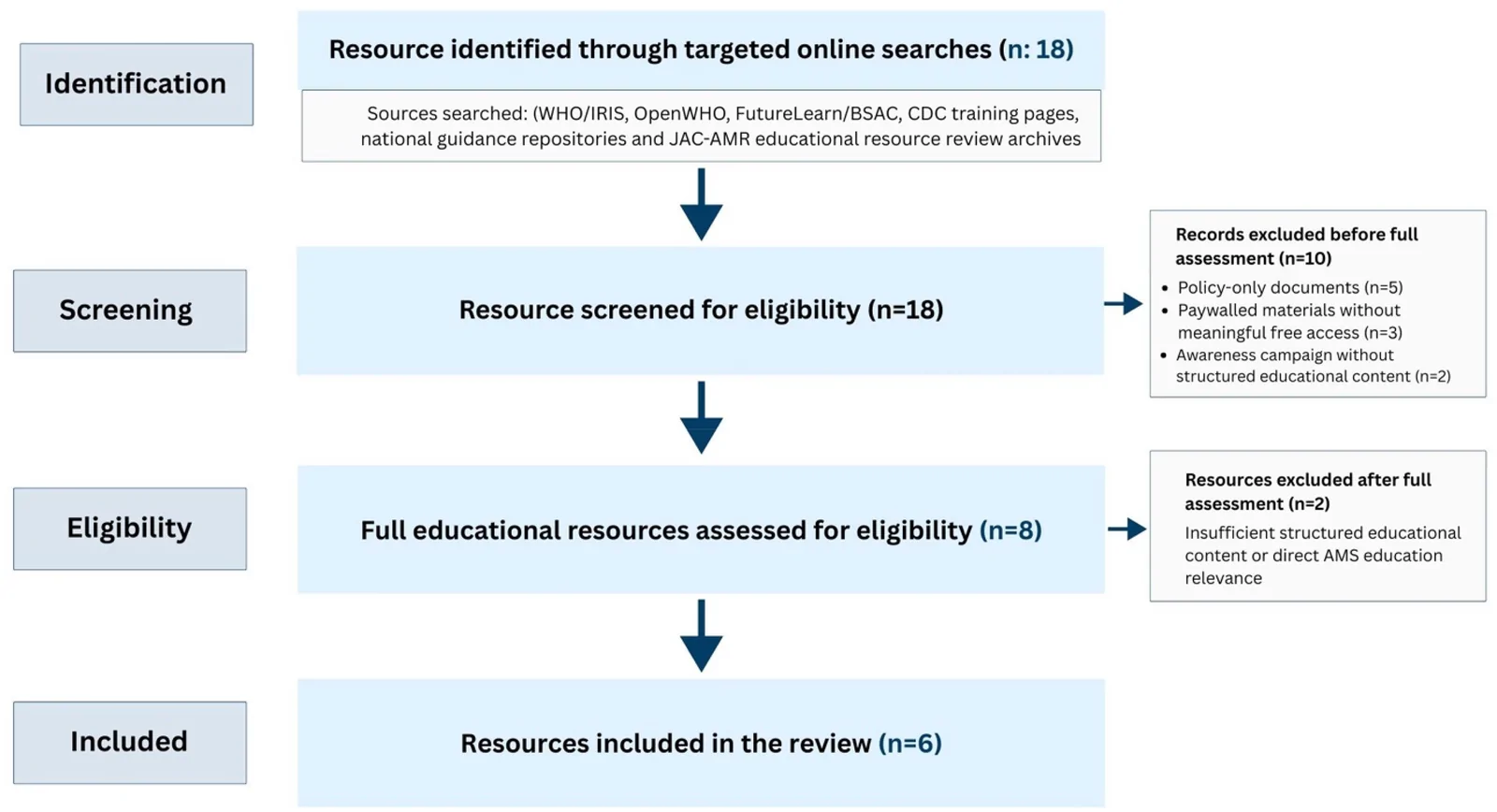
Resource identification and selection flow diagram. Targeted searches identified 18 records. After screening, policy-only documents (*n* = 5), paywalled materials without meaningful free access (*n* = 3), and awareness campaigns without structured educational content (*n* = 2) were excluded. Eight resources underwent full educational content assessment and six were included in the final review.

Resource identification strategy: The author team conducted targeted searches on 18 February 2026. Searches were planned around trusted sources likely to host AMS education for Ethiopia or adaptable LMIC settings, including national guidance repositories, WHO publications and Institutional Repository for Information Sharing (IRIS) entries, OpenWHO AMR course pages, FutureLearn stewardship MOOCs, CDC antibiotic stewardship training pages, BSAC resources and JAC-AMR educational resource review archives (Figure 1). Search terms combined: antimicrobial stewardship, AMS, antimicrobial resistance, AMR, Ethiopia, low- and middle-income countries, LMIC, education, training, toolkit, online course, MOOC, OpenWHO, FutureLearn, CDC, BSAC and WHO.

### Inclusion and exclusion criteria

Resources were included if they were publicly accessible online, explicitly educational in purpose, directly relevant to AMS practice and produced by credible institutions such as national ministries, WHO, universities, professional societies or public health agencies. Preference was given to resources designed for LMICs or adaptable to resource-limited settings.

Resources were excluded if they were policy documents without educational structure, paywalled materials without meaningful free access or awareness materials lacking structured learning content or practical stewardship relevance.

### Screening and selection procedures

Candidate resources were first screened using titles, landing pages, abstracts or resource descriptions, then reviewed in full when they appeared to meet the inclusion criteria. Final inclusion decisions were made by author consensus against the pre-specified eligibility criteria. Figure 1 summarizes the flow from 18 records identified to six included resources and lists the main reasons for exclusion.

### Data extraction

For each included resource, we extracted the title, web link, source organization, WHO region, country, World Bank income classification, format, anticipated duration, language, access model, accreditation or certification and downloadability.

### Appraisal framework

The appraisal framework was developed specifically for this review by combining the BSAC metadata classification with implementation-focused domains relevant to AMS education in Ethiopia. It was not a previously validated scoring tool. Domains were selected to capture whether resources were accessible, educationally usable, contextually appropriate, credible, current, stable, complementary and practically useful for moving from knowledge acquisition to AMS implementation. Each resource was appraised for accessibility, content scope, practical applicability, contextual relevance to Ethiopia, pedagogical design, credibility, bias, currency, platform stability, complementarity with other resources and overall value for AMS education and implementation.

## Results

**Figure 2.**
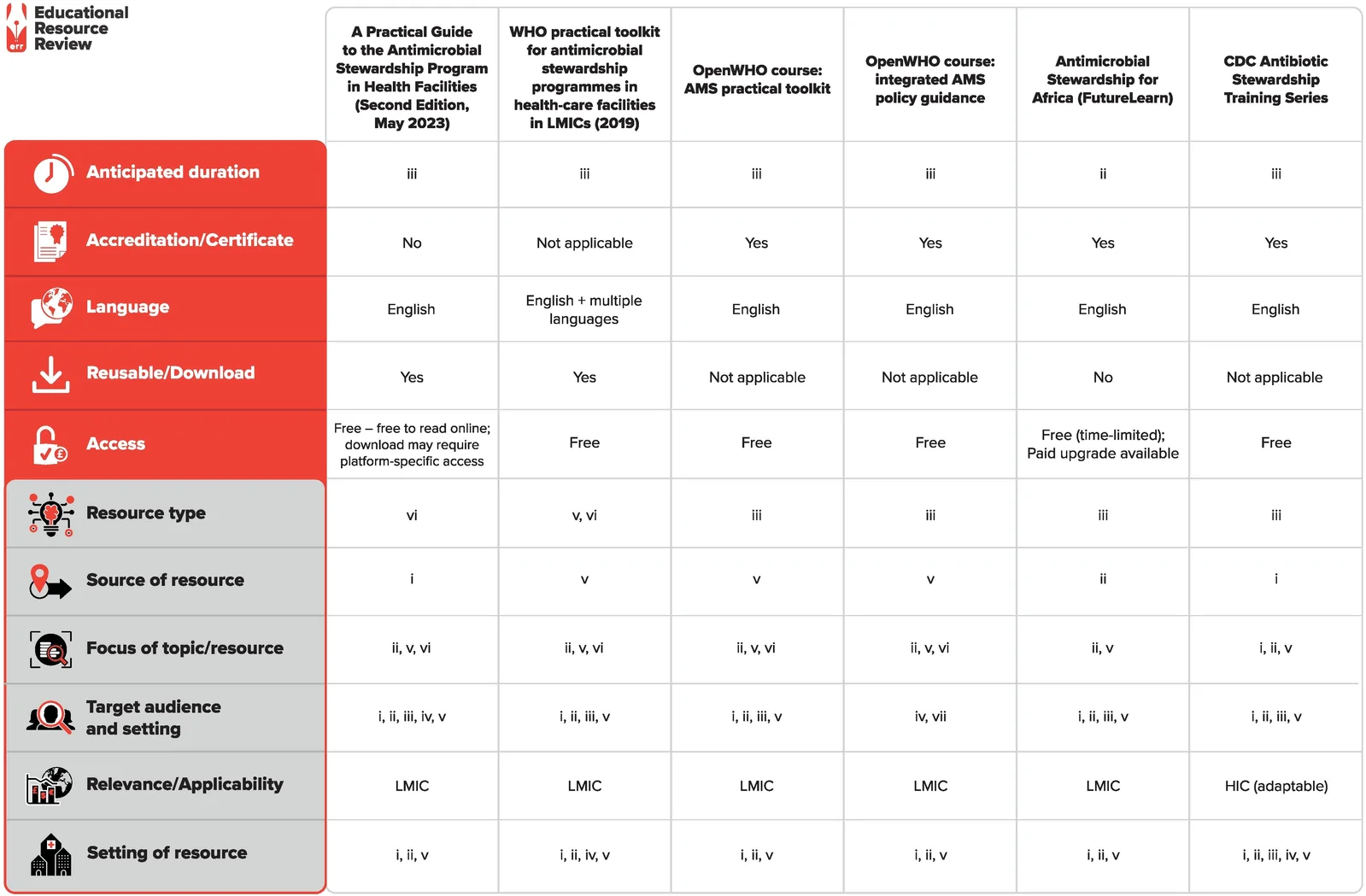
Characteristics of included online antimicrobial stewardship educational resources. Summary of six online antimicrobial stewardship (AMS) educational resources evaluated in this review, classified using a standardized educational resource framework across duration, format, source, topic focus, target audience, applicability, and care setting. Anticipated duration: (i) short duration (<4 h); (ii) long duration (>4 h); (iii) self-paced learning. Resource type: (i) website-based materials; (ii) news-oriented resources; (iii) online or distance learning courses; (iv) multimedia resources including webinars, videos, and podcasts; (v) clinical practice materials such as toolkits and guidelines; (vi) formal guidelines and policy documents; (vii) face-to-face course materials; (viii) e-books; (ix) public awareness materials; (x) commercial media; (xi) evidence-based resources; (xii) datasets and case studies; (xiii) patient narratives. Source of resource: (i) governments; (ii) professional societies; (iii) universities or higher education institutions; (iv) healthcare facilities; (v) international organizations; (vi) industry; (vii) insurance entities; (viii) non-governmental organizations. Focus of topic: (i) prudent prescribing; (ii) AMS principles and practice; (iii) infection management guidelines; (iv) infection prevention and control; (v) implementation and behaviour change; (vi) evaluation and measurement; (vii) evidence generation. Target audience: (i) doctors; (ii) pharmacists; (iii) nurses or midwives; (iv) non-medical managers; (v) public health professionals; (vi) laboratory personnel; (vii) general public; (viii) infection prevention practitioners. Applicability: (i) low-income countries; (ii) low- and middle-income countries; (iii) high- and middle-income countries; (iv) high-income countries. Setting: pre-service education and service settings including hospital, outpatient, community, long-term care, and mixed care environments.

Six resources were included, spanning national implementation guidance, a WHO LMIC toolkit, two structured OpenWHO e-learning courses (facility-level and national-level), an Africa-contextual MOOC and CDC stewardship trainings. In the World Bank fiscal year 2026 (FY26 table), Ethiopia is noted to be in a temporary unclassified status, which is relevant to how income-group applicability is reported for the national guide (Figure 2).

### Resource one: a practical guide to the antimicrobial stewardship program in health facilities (Second Edition, May 2023)

#### Description and scope

The guide frames AMS as a critical component of national patient safety and explicitly aligns itself with the National One Health AMR Prevention and Containment Strategic Plan (2021–2025).^2^ Specifically, it serves as the primary tool to implement the fourth strategic objective, optimizing the use of antimicrobials in human, animal and plant health care. The purpose section identifies a critical implementation gap by citing a national point prevalence survey (PPS) showing that none of the surveyed hospitals had functional AMS programmes, while inpatient antibiotic use was high at 63.8%. This data, derived from a multicentre study, provides the epidemiological rationale for the guide’s expanded scope, which now includes health centres in addition to hospitals.^3^

#### Accessibility

The resource was accessed via third-party platforms (e.g. Scribd, GH Supply Chain), which presents risks regarding version control and long-term stability. While the document is readable online, downloading requires a platform-specific sign-up, which may impede immediate access for frontline clinicians in resource-limited settings. The lack of a stable, direct download link from the official Ministry of Health portal is a notable barrier to national scale-up and reliable versioning.

#### Practical applicability and teachable components

The guide provides a highly actionable competency framework through its seven core elements: leadership commitment, accountability, pharmacy expertise, action, tracking, reporting and education. Notably, Section 2.2 adds Pharmacy Expertise as a distinct core element, recognizing the central role of clinical pharmacists in Ethiopian facilities. Teachable components include prospective audit and feedback (PAF), the WHO Access, Watch and Reserve (AWaRe) antibiotic classification and clinical toolkits.

#### Contextual appropriateness

The content is tailored to the Ethiopian health tier system. It specifies the multidisciplinary composition of the AMS committee and introduces the ‘ESCORT’ roadmap. This roadmap emphasizes a stepwise implementation strategy, advising facilities to ‘aim big but act small’ by targeting ‘low-hanging fruit’ interventions, such as ceftriaxone restriction or surgical prophylaxis audits, based on local feasibility and SWOT analysis.

#### Limitations

As an educational resource, the guide functions primarily as a technical handbook and administrative toolkit rather than a modular learning curriculum. It lacks built-in learner engagement mechanisms, such as interactive case studies, embedded assessments, or a structured syllabus for facilitator-led training. Furthermore, while the guide emphasizes ‘Education and Training’ as a core element, it does not provide the pedagogical materials (e.g. slide decks or pre/post-tests) required to verify workforce competency.

#### Judgement statement

This guide is an essential, high-utility resource for health facilities in Ethiopia, offering the most direct alignment with national clinical governance and the Strategic Plan (2021–2025). It provides a robust technical foundation for stewardship; however, for effective workforce development, it must be paired with a structured training curriculum, including local case scenarios and formal competency assessments, to bridge the gap between policy and practice.

### Resource two: antimicrobial stewardship programs in health-care facilities in low- and middle-income countries: a WHO practical toolkit (publication)

#### Description and scope

WHO’s 2019 practical toolkit is explicitly designed to support implementation of Global Action Plan objective four, optimizing antimicrobial use, by providing practical guidance for AMS programmes at national and facility level in LMICs.^4^ It targets national policymakers, hospital administrators and healthcare professionals, providing a stepwise framework for establishing AMS systems at both national and facility levels. It integrates both conceptual foundations and operational guidance, emphasizing doing and measuring AMS in real-world settings.

#### Accessibility

The toolkit is open access and freely downloadable from WHO’s website as a 71-page PDF document, which supports offline use in settings with intermittent connectivity. It is available in multiple languages (English, Arabic, French, Spanish and Russian), enhancing global usability. It is a self-paced reference resource, not requiring formal enrolment or payment.

#### Practical applicability

The toolkit is highly practical, offering step-by-step guidance for establishing and strengthening AMS programmes, supported by ready-to-use tools such as audit templates and monitoring frameworks. It emphasizes real-world implementation in resource-limited settings, making it directly applicable for healthcare facilities. This practical sequencing closely matches the Ethiopian guide’s stepwise governance and monitoring and evaluation (M&E) orientation, suggesting strong complementarity. However, effective use depends on institutional support and baseline AMS knowledge.

#### Contextual appropriateness for Ethiopia

The toolkit’s explicit LMIC focus and its intent to operationalize national action plan implementation make it a strong fit for Ethiopia’s national strategy emphasis. Its flexible, scalable approach allows adaptation across different levels of the Ethiopian healthcare system. However, some recommendations may require local tailoring to align with national guidelines and resistance patterns.

#### Limitations

As a document-based toolkit, engagement depends on facilitation. If used without structured teaching, the learning burden is high and may not translate into behaviour change, consistent with broader evidence that education alone may not sustain AMS improvements.

#### Judgement statement

A high-value offline reference that can standardize AMS programme design and measurement across facilities, but impact depends on converting toolkit content into teachable, assessable local training packages aligned to Ethiopian workflows.

### Resource three: OpenWHO course based on the WHO LMIC AMS practical toolkit

#### Description and scope

This self-paced OpenWHO course translates the WHO LMIC toolkit into structured learning with modules and a guided simulation.^8^ It is designed to provide practical, step-by-step guidance on establishing, implementing and monitoring AMS programmes within healthcare facilities, specifically tailored for the contexts of low- and middle-income countries (LMICs). It complements the national-level guidance provided in the accompanying policy course.

#### Accessibility

The course is highly accessible, hosted for free on the OpenWHO platform. A certificate (Record of Achievement) is awarded upon successful completion of the graded assessments. A critical platform-level risk is that OpenWHO states it is changing to a hub model and will not provide certificates going forward, which can reduce incentives for learners and complicate national CPD integration.

#### Educational design and engagement

The course is well-structured, dividing content into seven focused modules followed by a summary and an assessment. This modular design may help reduce learner fatigue. While the core content includes text-based presentations, it is supplemented with downloadable slides, and crucially, a guided simulation. The inclusion of a final, graded quiz provides an objective measure of knowledge retention

#### Contextual appropriateness for Ethiopia

The course is explicitly tailored for LMIC health-care settings and includes stepwise, low-resource implementation advice that is readily adaptable to Ethiopian hospitals and regional referral centres. It provides a common, WHO-endorsed framework that can be used to standardize AMS education and implementation efforts across the country.

#### Limitations

Reliance on stable internet and a learning platform may limit reach in facilities outside major cities. Platform transition and certificate uncertainty are major implementation risks for workforce credentialing pathways. Additionally, while the course mentions the skills needed for an AMS team, it is not a substitute for advanced clinical or leadership training.

#### Judgement statement

High educational structure and assessment value for facility AMS teams, but scalability in Ethiopia requires low-bandwidth or offline packaging and policy decisions on credential recognition given OpenWHO platform changes.

### Resource four: OpenWHO course on WHO policy guidance for integrated national AMS activities

#### Description and scope

A policy-level OpenWHO course translating the WHO Policy Guidance on integrated national AMS activities into learning modules and assessments.^9^ It covers national coordination, the package of integrated AMS activities, implementation considerations and a periodic national assessment tool. It is designed for national-level implementers and programme planners.

#### Accessibility

Like its counterpart, this course is freely available on the OpenWHO platform and potentially useful for policymakers and health officials, including in Ethiopia. A certificate is awarded upon successful completion of the graded assessments. The same OpenWHO platform transition risk applies, including the stated shift to a hub model without certificates.

#### Educational design and engagement

Module structure is concise and policy oriented. Learning materials include lecture content, topic subpages, a guided simulation and an objective assessment. Engagement is appropriate for policymakers and programme managers but is less clinical and less case-based than some facility-level AMS courses.

#### Contextual appropriateness for Ethiopia

Highly relevant for Ethiopian national AMR planners, ministries of health and multisectoral AMR committees. The course emphasizes national coordination, surveillance, Infection Prevention and Control and Water, Sanitation, and Hygiene (IPC/WASH) links and resource mobilization, key areas for Ethiopia’s national action plan implementation. The Periodic National Assessment Tool is particularly useful for baseline assessment and planning.

#### Limitations

The course’s primary limitation is its target audience; it is less suited for individual clinicians or facility-level staff. Its utility depends on the existence of national and regional planning capacity and may not translate into daily prescribing changes without downstream facility training and audit systems.

#### Judgement statement

Strongest option in the set for national AMS coordinators and planners, but it should be implemented as part of a national cascade training model that links national planning competencies to facility-level tools and supervision.

### Resource five: ‘Antimicrobial Stewardship for Africa’ MOOC (FutureLearn, developed by BSAC and partners)

#### Description and scope

‘AMS for Africa’ is a MOOC developed by the BSAC in collaboration with partners including the Infection Control Africa Network and the University of Lagos College of Medicine.^10^

The course is structured as a three-week programme that requires an estimated three hours of study per week and provides a digital certificate to eligible learners.

It covers key topics including AMR epidemiology, prescribing optimization, infection prevention and control (IPC) and development of stewardship programmes and policies. The course integrates case-based examples and regional perspectives to support practical application in low- and middle-income countries (LMICs).

#### Accessibility

The course is delivered online via the FutureLearn platform and is globally accessible in English. Participation is free for a limited time, while extended access and certification require payment. This time-limited access model may affect completion rates and equitable participation in resource-constrained settings.

#### Target audience

The course is intended primarily for healthcare professionals involved in infection prevention and antimicrobial prescribing, including physicians, pharmacists, nurses, microbiologists and public health practitioners. It may also be relevant to policymakers and healthcare managers developing AMS strategies.

#### Educational design and engagement

The course follows a MOOC model, using short videos, articles, case studies and discussion forums to support interactive and peer-based learning. It emphasizes a social learning approach, allowing participants to share experiences and perspectives on AMS.

The content focuses on AMR in Africa, including its burden and strategies to reduce inappropriate antibiotic use. It also covers key areas such as developing antibiotic policies, implementing hospital-based AMS programmes and applying behavioural approaches to improve prescribing. Infection prevention and control (IPC) and regional collaboration are also introduced.

By the end of the course, learners are expected to assess AMS strategies in African healthcare settings, identify practical prescribing improvements in low-resource environments and develop context-appropriate antibiotic guidelines and policies.

#### Contextual appropriateness for Ethiopia

The course is highly relevant to Ethiopian healthcare settings due to its focus on African case studies, resource constraints and behavioural drivers of prescribing. Its emphasis on multidisciplinary teamwork, guideline development and IPC aligns well with national AMS priorities and can support capacity building across hospitals and regional facilities.

#### Limitations

Dependence on stable internet access may limit reach outside major urban centres. The time-limited free access and paid certification model may restrict widespread uptake. Additionally, variability in user experience and platform navigation may affect learner engagement.

#### Judgement statement

High contextual relevance and strong engagement through social and case-based learning make this a valuable AMS resource for Ethiopia. However, its impact in Ethiopia may be constrained by connectivity requirements and platform access limitations, suggesting that the course may be most effective when delivered through institutional support.

### Resource six: CDC antibiotic stewardship trainings and training series

#### Description and scope

The ‘CDC Antibiotic Stewardship Training Series’ is a self-paced training series developed by CDC, consisting of nine modular online units designed to strengthen knowledge and clinical practice in AMS.^11,12^ It covers core topics such as AMR, healthcare team roles, prescribing optimization and communication strategies, supported by lectures, case discussions. Optional continuing education credits are available for participants.

#### Accessibility

The training is freely available online through the CDC TRAIN platform and can be accessed globally. Its flexible, self-paced format allows learners to complete modules in any order, supporting adaptability for different learning needs and schedules.

#### Target audience

The training series is primarily intended for healthcare professionals involved in antibiotic prescribing and stewardship activities. This includes physicians, nurse practitioners, pharmacists, nurses and public health professionals. The content is particularly suitable for clinicians seeking foundational knowledge in antibiotic stewardship or those participating in institutional stewardship programmes.

#### Educational design and engagement

The programme follows a structured, modular design aligned with adult learning principles, incorporating knowledge-check quizzes and case-based examples to reinforce learning. However, the approach is predominantly lecture-based, with limited interactive or simulation-based components, which may reduce engagement and deeper learning.

#### Contextual appropriateness for Ethiopia

The training provides foundational AMS principles that are broadly applicable in Ethiopia, particularly in areas such as prescribing behaviour and clinician-patient communication. However, much of the content is based on the US healthcare context, which may limit direct applicability without adaptation to local resource constraints, disease patterns and health system structures.

#### Limitations

A key limitation is the strong focus on epidemiological data, clinical examples and healthcare systems specific to the USA, which may reduce relevance in LMIC settings.

In addition, the lecture-based format provides only moderate engagement and may not offer enough advanced content for experienced professionals. To be effective in Ethiopia, the training would need to be adapted to reflect local disease patterns, diagnostic capacity, availability of antimicrobials and regulatory systems.

#### Judgement statement

Overall, the CDC Antibiotic Stewardship Training Series is a credible and accessible educational resource for foundational AMS training. Its modular design and focus on prescribing behaviours and communication make it potentially valuable for capacity building; however, careful contextual adaptation and selective use of modules are necessary to ensure alignment with Ethiopia’s healthcare context and AMS priorities.

## Discussion

### Key findings and synthesis across resources

**Figure 3.**
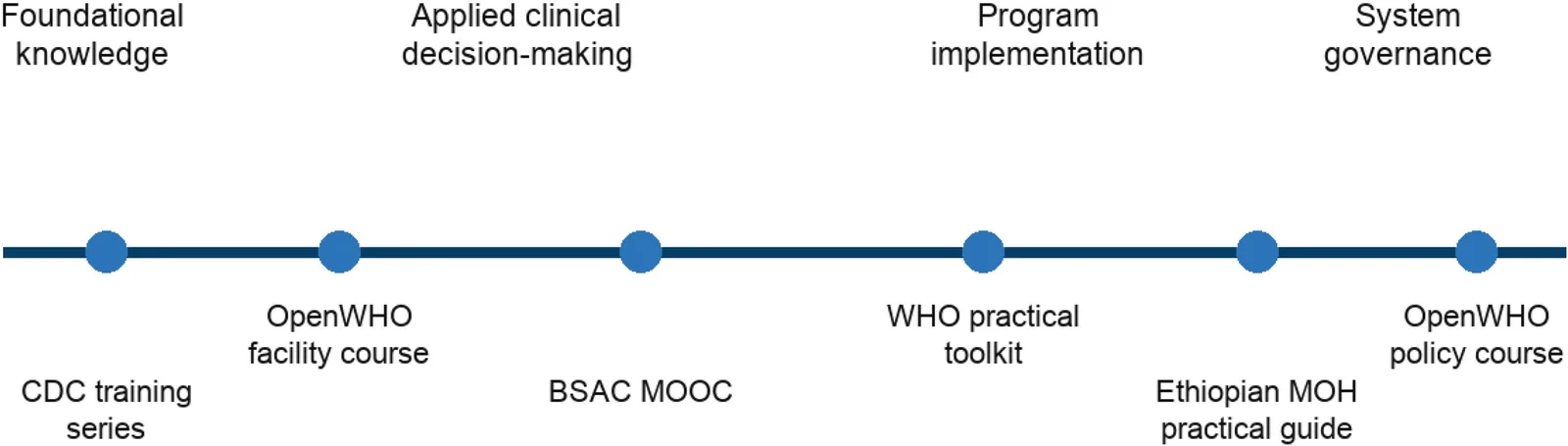
Positioning of included online AMS resources across the education-implementation continuum. Conceptual mapping of the six included resources from foundational knowledge to system-level governance. The CDC training series, OpenWHO facility course, BSAC MOOC, WHO practical toolkit, Ethiopian Ministry of Health practical guide and OpenWHO policy course are positioned according to their dominant educational function.

This review identified a clear hierarchy of educational and implementation utility across the six included resources (Figure 3).^2,4,8–11^ The Ethiopian national AMS practical guide was the most directly relevant resource for local use because it aligned closely with national priorities and provided the greatest operational detail.^2^ In contrast to more generic educational materials, it addressed stewardship governance, committee composition, core elements, intervention selection, audit and feedback, AWaRe integration and IV-to-oral switching within the realities of the Ethiopian health system.^2^ This made it the strongest resource for translating policy into facility-level action.

The WHO LMIC practical toolkit complemented the Ethiopian guide by offering a standardized framework for AMS design, implementation and monitoring that is applicable across low- and middle-income settings.^4^ Its value lies in its structured and programmatic approach, which can support consistency across institutions. However, like the Ethiopian guide, it functions primarily as a reference document and depends on facilitated use if it is to drive meaningful educational outcomes.

The two OpenWHO courses addressed this limitation by converting stewardship guidance into modular, self-paced learning with structured content and assessment.^8,9^ These resources offered better educational design than document-based toolkits and are especially useful for standardizing foundational AMS training. However, their dependence on stable internet access and uncertainty around future certificate provision may limit their long-term value for national credentialing and continuing professional development.

The Africa-focused MOOC added an important regional dimension by incorporating African case examples, stewardship challenges and behavioural perspectives that are more relatable to Ethiopian learners than materials developed primarily for high-income settings.^10^ Its social learning design and case-based format strengthen engagement.^13^ Nevertheless, its reliance on internet access and platform-based participation may reduce reach, especially outside major urban centres.

The CDC training series contributed credible and well-structured modules on stewardship principles, prescribing behavior and communication.^11^ These are useful for foundational learning, particularly in areas such as clinician–patient interaction and antibiotic decision-making.^12^ However, the strong US orientation of the content limits direct transferability, and selective adaptation would be necessary before it could be recommended widely for Ethiopian practice.

Taken together, these findings suggest that no single resource is sufficient on its own. The most effective approach for Ethiopia is likely to be a blended educational model that combines the national practical guide as the implementation anchor,^2^ WHO and OpenWHO materials as structured learning supports, and regionally relevant case-based resources to strengthen clinical reasoning and behaviour change. This aligns with evidence from virtual communities of practice in sub-Saharan Africa, which demonstrate that structured, case-based educational interventions can successfully drive behaviour change and strengthen healthcare systems in low-resource settings.^14^

### Integration with Ethiopia’s national AMS strategy and workforce development

A major strength of this resource set is that several materials can be mapped directly onto Ethiopia’s national AMR and AMS priorities. The Ethiopian practical guide explicitly positions stewardship as a mechanism for implementing the national strategy to optimize antimicrobial use.^2^ It also responds to a clear systems gap, namely the absence of functional AMS programmes in health facilities and the high prevalence of inpatient antibiotic use. These realities indicate that AMS education in Ethiopia must go beyond awareness and focus on practical workforce capacity. Studies from Ethiopian hospitals confirm that while health professionals recognize AMR as a significant problem, formal ASPs are largely non-functional, and there is an urgent need for education, institutional guidelines and prospective audit with feedback interventions.^15^

At least three levels of competency development emerge from the included resources. First, national and regional leadership capacity is best supported by the OpenWHO course on integrated national AMS policy guidance,^9^ which addresses coordination, planning, surveillance and national assessment. Second, facility-level governance and implementation are best supported by the Ethiopian practical guide together with the WHO LMIC toolkit, both of which provide stepwise approaches to committee formation, intervention planning and monitoring. Third, frontline prescribing behaviour can be strengthened through the practical tools in the Ethiopian guide, supported by case-based learning from the Africa MOOC and selected communication-focused content from the CDC modules.

This layered approach is important because education alone is unlikely to produce sustained stewardship gains unless it is linked to mechanisms that reinforce practice change.^7^ The Ethiopian guide is particularly valuable in this regard because it connects learning with tracking, reporting, feedback and quality improvement.^2^ For Ethiopia, the most useful online educational resources are therefore those that combine knowledge development with tools that embed stewardship into routine care.

Pedagogically, these findings favour blended and work-based approaches rather than stand-alone online modules. For Ethiopia, the strongest options are likely to combine short didactic content with facilitated case discussions, local audit-and-feedback exercises, peer learning or communities of practice and practical assignments linked to existing facility AMS committees. This approach is consistent with evidence that work-based AMS education can improve practice when it is tied to local workflows,^7^ with virtual communities of practice in sub-Saharan Africa that supported case-based learning and implementation support,^14^ and with Ethiopian evidence on pharmacist-led educational and audit-oriented stewardship interventions.^15^ Where internet access is unreliable, the same content could be delivered through downloadable slide sets, printed facilitator guides, offline microlearning packages, face-to-face CPD workshops and locally hosted resource libraries.

### Gap analysis

**Figure 4.**
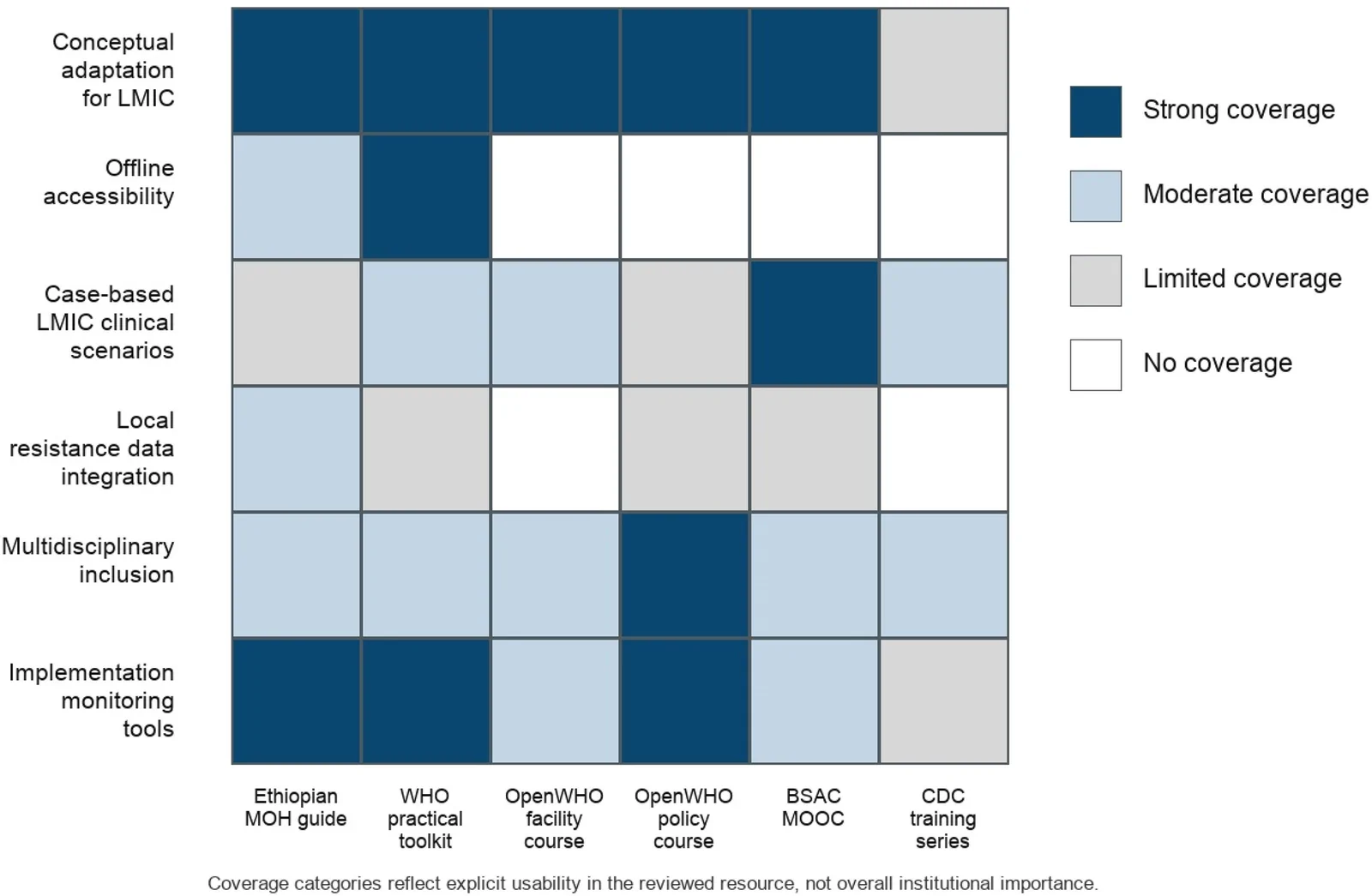
Gap analysis of online AMS educational resources relevant to Ethiopia. Matrix-based comparison of the six included resources across contextual adaptation for LMIC settings, offline accessibility, case-based clinical scenarios, local resistance data integration, multidisciplinary inclusion and implementation monitoring tools. Strong coverage indicates explicit, directly usable content; moderate coverage indicates relevant content requiring local adaptation or facilitation; limited coverage indicates indirect or minimal coverage; no coverage indicates that the domain was not meaningfully addressed.

Three major gaps were evident across the included resources (Figure 4).

First, there is a substantial offline access gap. The most structured learning resources, particularly OpenWHO and the MOOC platform, depend on stable internet connectivity and digital access.^13,16^ This creates an equity problem for facilities and learners in lower-resource or geographically remote settings. By contrast, the Ethiopian guide and WHO toolkit are more usable offline, but they are less interactive and depend more heavily on local facilitation.

Second, there is a language gap. Most resources are available primarily in English. Although some WHO materials are translated into multiple international languages, localized African language availability remains limited.^9^ This may reduce accessibility for parts of the health workforce and may constrain large-scale educational uptake if materials are not adapted into more locally usable formats.

Third, there is a gap in Ethiopia-specific clinical training scenarios. The national guide is contextually strong at the policy and implementation level,^2^ but scalable educational delivery would benefit from case-based modules that reflect local diagnostic limitations, antimicrobial availability, resistance patterns and prescribing pressures. This is particularly important in LMIC settings, where stewardship decisions often need to be made under conditions of uncertainty, limited microbiology access and inconsistent medicine supply.^6^

### Recommendations for educators, policymakers and resource developers

**Figure 5.**
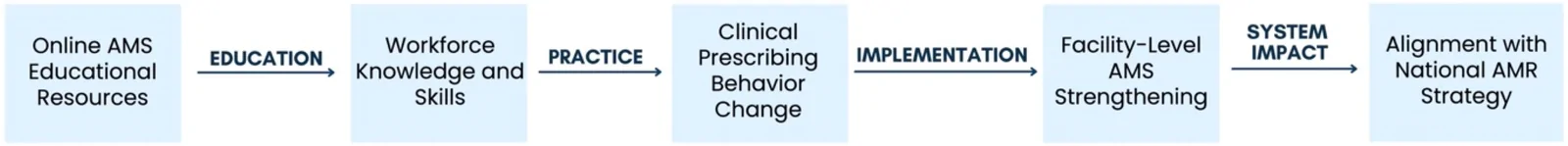
Digital antimicrobial stewardship education pathway for Ethiopia. Conceptual model illustrating the progression from online AMS educational resources to workforce knowledge acquisition, clinical prescribing behaviour change, facility-level stewardship strengthening, and alignment with national antimicrobial resistance strategy. Grouped domains represent stages of impact from education to system-level outcomes, emphasizing the role of integrated and context-adapted learning approaches.

For educators, the most practical next step would be to build a blended national AMS curriculum using the Ethiopian practical guide as the core implementation document and supplementing it with selected online courses for structured teaching (Figure 5).^2^ Short competency-based modules could be built around audit and feedback, AWaRe use, IV-to-oral switching and stewardship committee functions. Evidence from pharmacist-led educational interventions in Ethiopia suggests that such approaches are feasible and well-received by health professionals.^2^

For policymakers and programme leaders, priority should be given to stable and scalable delivery mechanisms. Reliance on external platforms whose certification systems may change introduces risk for national continuing professional development pathways. Downloadable learning packages, nationally hosted training materials and formal alignment with local accreditation systems would strengthen sustainability.

For resource developers, the most valuable additions would be offline-capable microlearning formats, locally relevant case libraries, practical facilitator materials and concise translated summaries for multidisciplinary teams. Educational resources are likely to have greater impact when they are explicitly linked to routine implementation tools, especially those already embedded in the Ethiopian guide.^2^

An additional equity consideration is the temporary unclassified status of Ethiopia in the World Bank FY26 income grouping.^17^ This reinforces the need to avoid simplistic assumptions about resource availability and supports the case for low-cost, resilient and adaptable educational delivery models.

### Limitations

This review has several limitations. First, it used a targeted search strategy across major trusted platforms rather than a fully systematic search, so some relevant resources may have been missed. Second, online educational resources are dynamic and may change over time in terms of access, hosting, certification or content. Third, the Ethiopian practical guide was accessed through third-party hosting rather than a stable official Ministry of Health repository, which creates concerns regarding version control and long-term accessibility. These limitations should be considered when interpreting the findings and when planning future national use of these resources.

### Conclusions

These six online AMS educational resources offer complementary value for Ethiopia, with the national practical guide providing the strongest implementation relevance and the WHO- and course-based resources strengthening structured learning and broader stewardship capacity. A blended, context-adapted approach that prioritizes offline access, local case-based training and alignment with national AMS strategy is likely to provide the greatest educational and system-level impact.
